# Digital Leadership and Employee Creativity: The Role of Employee Job Crafting and Person-Organization Fit

**DOI:** 10.3389/fpsyg.2022.827057

**Published:** 2022-05-09

**Authors:** Jian Zhu, Bin Zhang, Mingxing Xie, Qiuju Cao

**Affiliations:** ^1^Business School, Xiangtan University, Xiangtan, China; ^2^School of Humanity, Shanghai University of Finance and Economics, Shanghai, China; ^3^Economic and Trade School, Guangxi University of Finance and Economics, Nanning, China

**Keywords:** digital leadership, employee job crafting, person-organization fit, employee creativity, moderated mediating model

## Abstract

Industry 4.0 has changed the paradigm in the business practice and business model, and digital technology has brought radical transformations to enterprises. To support this transformation, digital leaders are required to help enterprises transform and lead them to a more promising future. Based on job demands-resources model and person-organization fit theory, this study examines the relationship between digital leadership and employee creativity. Based on a sample of 357 employees from various Chinese companies, this study used SPSS 22.0 and MPLUS 7.0 to examine the hypotheses. The findings indicate the following (a) digital leadership has a positive effect on employee creativity. (b) employee job crafting mediate the relationship between digital leadership and employee creativity. (c) person-organization fit positively moderates the relationship between digital leadership and employee job crafting. (d) person-organization fit positively moderates the indirect effect of digital leadership on employee creativity *via* employee job crafting. The findings reveal the effect mechanism of digital leaders on employee creativity and enrich the literature on antecedents of employee creativity. Practical implications and future research are also discussed.

## Introduction

Industry 4.0 has given great impetus to the change in paradigm in the business practice and business model, dominated by digital technologies (Mihardjo et al., 2019). In pace with the development of technologies in companies, such as mobile Internet, cloud computing, artificial intelligence, big data, the Internet of things, and blockchain, executives are increasingly facing various challenges associated with digitalization. Particular challenges are the simultaneous handling of many topics, considerable information flushing *via* digital channels, rapid changes, and finding the optimal balance between the old and the new (Temelkova, 2018; Klus and Müller, 2021). For leaders, digital technologies mean new forms of communicating and organizing (El Sawy et al., 2016), but classic leadership styles do not sufficiently address the opportunities and challenges arising from digitalization (Klein, 2020). In conditions of significant shifts in the digital era, amid the social and technological environment, modern business organizations are increasingly requiring a new type of leadership able to thrive in a digital environment and that is characterized by high-tech skills leading to optimal management and optimal team collaboration (Abbu and Gopalakrishna, 2021; Bresciani et al., 2021). Driven by the increasing influence of technology on leadership, digital leadership has been put forward emphasized. Digital leadership is defined as the leaders’ ability to create a clear and meaningful vision for the digitalization process and the capability to execute strategies to actualize it (Larjovuori et al., 2016; de Araujo et al., 2021).

The existing literature of digital leadership concentrates on its effects at the macro level such as business mode innovation, innovation management, and dynamic capability (Wasono and Furinto, 2018; Mihardjo et al., 2019; Promsri, 2019; Sasmoko et al., 2019). Sasmoko et al. (2019) argued that digital leadership is positively related to innovation capability (Sasmoko et al., 2019). Wasono and Furinto (2018) argued that enterprises would increase sustainable competitive advantage in the disruptive era through strengthening the digital leadership and innovation management (Wasono and Furinto, 2018). Mihardjo et al. (2019) suggested that digital leadership had direct and indirect impacts effects on customer experience orientation in developing business model innovation (Mihardjo et al., 2019). Soon and Salamzadeh (2021) proposed that digital leadership has a great impact on virtual team effectiveness. However, organizational growth depends on the ability of generating to generate novel ideas and implementing those that are promising and feasible novel ideas. In short, creativity (the generation of the novel ideas) and innovation (the implementation of the novel ideas) are vital for the organization to survival survive and succeeds (Anderson et al., 2014). Unfortunately, it is rare in literature studies that regard digital leadership as a key antecedents to predict employee creativity at the micro level are rare. Thereby, given the widespread adoption of network organization in most areas of business and the importance of employee creativity, it is vital to examine how and when digital leadership affects employee creativity. To address this theoretical gap, this study constructs a theoretical model to test the influence of digital leadership on employee creativity. As the job demands-resources model proposed, job characteristics can be categorized into two broad classes: job demands or job resources (Bakker and Demerouti, 2007). Job resources can buffer the effect of job demands on exhaustion, whereas job demands strengthen the motivating role of job resources (Demerouti et al., 2001). Consequently, individuals could seek resources and optimize demands to enhance the pool of resources and help deal with job demands by optimizing their burden (Bakker and Demerouti, 2007). On the basis of job demands-resources model, we argue that job crafting will mediate the relationship between digital leadership and employee creativity. On the one hand, digital technology will improve the flexibility of the organization and the utilization of resources to some extent by empowerment (Abbasi et al., 2020). On the other hand, digital technology has changed the traditional way of work (Gilson et al., 2015). Therefore, employees will make job crafting to enable their abilities to fit their jobs, and then lead to enhance their creativity. That is to say, digital leadership may indirectly affect employee creativity via employee job crafting.

Moreover, person–organization fit theory assumes that is attitudes, behavior and other person level outcomes result not from the person or the organization independent of each other, but rather from the relationship between the person and organization (Westerman and Cyr, 2004; Morley, 2007; Johnson et al., 2014). Person–organization fit is categorized into two broad classes: supplementary fit (measured by values congruence and personality congruence), and complementary fit (measured by work environment congruence) (Kristof, 1996). Supplementary fit is typically assessed as similarity on psychological characteristics such as values, goals, attitudes, or personality traits, whereas complementary fit often refers to a person possessing the requisite knowledge, skills, and abilities to meet job demands (Muchinsky and Monahan, 1987; Kristof-Brown et al., 2014; Seong et al., 2015). Person-organization fit has been proved to be related to behavior outcomes, including organizational commitment, organizational citizen behavior (Wei, 2012), job performance (Hoffman and Woehr, 2006), and innovative work behavior (Afsar, 2016). Accordingly, high levels of person–organization fit can enhance the relationship between digital leadership and employee job crafting.

Overall, this study constructed a model to test how and when digital leadership affects employee creativity by integrating the job demands–resources model and person–organization fit theory. This study makes three contributions. First, by investigating the influence of digital leadership on employee creativity, this study not only enriches the research on leadership, but also explores the key antecedents of employee creativity. Second, this study examines the mediating effect of employee job crafting, that is, digital leadership indirectly relates to employee creativity through employee job crafting. Third, this study extends the boundary conditions under which the mediating effect of employee job crafting between digital leadership and employee creativity are strengthened or weakened.

## Theory and Hypotheses

### Digital Leadership

Enterprises have entered an age marked by rapid business, in organizational culture, and corresponding tensions between “change makers.” Therefore, organizations have become more flexible and create a decentralized workplaces for members to achieve the expectation of higher productivity (Kane et al., 2019). Driven by the increasing influence of technology on leadership, a growing body of digital leadership has developed that draws on well-established human resources studies, but also takes new directions. Different ages have required different leadership styles. Throughout history, technological transformation has been shaping different types of leadership. Therefore, leadership has developed differently based on different patterns, such as hierarchy, power, authority and personality. However, in digital economy, digitization has significantly changed leadership styles and skills (de Araujo et al., 2021), and poses new challenges to leaders. They need to adapt to the uncertain environment and enhance their digital knowledge to lead the companies effectively. To meet increasingly complex and changing demands, the concepts of digital leadership have emerged as the most relevant leadership styles.

Following prior work, we define digital leadership as the leaders’ ability to create a clear and meaningful vision for the digitalization process and the capability to execute strategies to actualize it (Larjovuori et al., 2016). Digital leadership is regarded as a fast, cross-hierarchical, team-oriented, and cooperative leadership style, that keeps a strong focus on an organization’s innovation (Oberer and Erkollar, 2018). Recently, a large and growing body of literature has explored the characteristics of digital leadership. Klein (2020) argued that digital leaders have creative thinking, foresight and insight. According to Larjovuori et al. (2016), digital leadership has distinctive characteristics, such as creativity, in-depth knowledge, strong network and collaboration, and loyal participation *via* vision. Based on these studies, the digital leadership in this study considers a number of factors, including creativity, deep knowledge, global vision and collaboration, thinking, inquisition continual learning and sensitivity to digital opportunities. In line with Kane et al. (2019), we hold the points that leaders need to have four key skills in digital age: transformational vision, forward-looking perspective, digital literacy and adaptability. First, providing vision and direction for enterprises and employees has long been an important part of leadership, but digital leadership have a new significance by placing more emphasis on controlling future changes. Leaders with a transformational vision can more effectively predict markets and trends, make smart business decisions, and solve difficult problems in turbulent times (Luck et al., 2012). Second, leaders with a forward-looking perspective will have a clear vision and reasonable strategy, and can grasp the trend in the digital trend (Klein, 2020). Third, digital literacy can support the first two skills. Leaders who lack digital knowledge hinder the development of emerging technologies and trends. A possible reason for this limitation that they can’t control both the value brought by emerging technologies and the threat to the organization. Finally, When the market and technology environment is full of unknown and uncertainty, adaptive performance helps organizations find a key way forward. This mentality can also promote leaders to update their professional knowledge and skills in time to ensure that they keep pace with the times.

### Digital Leadership and Employee Creativity

The economy in the digital age is mainly manifested in high-level digitization, major technological progress and innovation. Therefore, enterprises need to produce and provide high value-added products and services, obtain competitive advantages over competitors, and optimize management processes (Temelkova, 2018). Therefore, digital technology calls for a change in the role of leaders. Leaders need to have new skills to help their organization effectively deal with the uncertainty and complexity of the environment and lead organizations to a more dynamic future (de Araujo et al., 2021).

Digital leadership plays an important role in promoting employee creativity (Wasono and Furinto, 2018; Mihardjo et al., 2019). Employee creativity is defined as the generation of new and useful ideas for the organization, including new products, ideas, services, and management methods (Baer and Oldham, 2006). In the era of digital economy, digital technology not only redefines leadership, but also has a far-reaching effect on organizations and employees (Hensellek, 2020). Specifically, on the one hand, digitization is a process of constant change with an open outcome. Organizations are required to be more flexible and constantly adjust at all part of the organization. Owing to digitalization, leaders need to realize that their work environment and demands are changing. They no longer simply assign tasks to their subordinates and monitor the completion of tasks, but also involveed in creating space for the development of team members’ creative potential through collaboration and continuous learning (Bass and Riggio, 2006). The digital transformation of enterprises also promotes leaders’ digital mindset, and they must be able to effectively and efficiently integrate digital technologies into the day-to-day work of themselves and their employee (Hensellek, 2020). Digital leader are willing to set a good example and take responsibility for digitalization efforts to signal his/her commitment to mitigating the inherent uncertainties of digitalization.

On the other hand, in pace with the development of technologies in companies such as mobile Internet, cloud computing, artificial intelligence, big data, the Internet of things, blockchain, and other technologies in companies (Temelkova, 2018), digital technology has changed the traditional way of work. Using digital tools, executives can establish remote workplaces and virtual teams to complete tasks by independence from a given time and place as well as changing work demands in general (Gilson et al., 2015). The emergence of WeCom, DingTalk, and email increasingly facilitates the employees’ communication and knowledge sharing within organizations, and promotes their job autonomy and creativity. In summary, digital leaders are able to articulate an explicit transformational vision and forward-looking perspective for a digital future, and have both the positive attitude and the necessary skills to stimulate employee creativity.

Digital leadership can also help enterprises formulate future development plans, initiate sustainable changes and promote enterprise performance by accessing to the latest technical information and establishing appropriate relationships (Larjovuori et al., 2016). Digital technology has fundamentally changed the way people communicate and interact with each other and the way companies operate in the market. The leadership function has also changed from the traditional “command and control” to “communication and cooperation” (Gozman and Willcocks, 2019). Given that information is becoming open in the Internet and digital era, everyone has the ability to obtain, process and apply information, and apply digital assistant technology to better complete tasks (Wasono and Furinto, 2018). Therefore, digital leadership not only needs to master the latest technical knowledge at all times and set an example for employees to learn continuously, but also provide appropriate authorization to create an atmosphere supporting innovation for the team. Team members will adjust their working methods to adapt to the changing environment, better complete work tasks and stimulate their creativity based on leadership behavior and supporting innovation atmosphere.

***Hypothesis 1:** Digital leadership is positively related to employee creativity.*

### Digital Leadership and Employee Job Crafting

Employee job crafting can be regarded as a bottom-up approach to job redesign in which employees could find work meaning and fit their organization by altering their jobs (Demerouti et al., 2021). In this study, we conceptualize employee job crafting from the perspective of the job demands-resources model. Accordingly, job crafting represents the changes that employees make to balance their job demands and job resources with their personal abilities and needs (Tims et al., 2012). Employees can change their jobs in many ways. Examples include adjusting the scope of activities executed at work, changing with whom they work, refreshing job meaning, making changes to the knowledge and skills required by their work, or avoiding interaction with unpleasant clients (Meijerink et al., 2020). Through job crafting, employees can improve their work to fit their individual skills, needs, and preferences (Tims and Parker, 2020).

The job demands-resources model was first proposed by Demerouti et al. (2001) in, and has been gradually improved through the continuous development of scholars. According to the job demands-resources model, all job characteristics can be categorized into two broad classes: job demands and job resources. Job demands represent all aspects of the job that require sustained physical and/or psychological (cognitive and emotional) effort or skills (Bakker and Demerouti, 2007; Demerouti et al., 2021). Therefore, job demands are associated with certain physiological or psychological costs and work stress. Job resources refer to those aspects of the job that are either/or functional in achieving work goals. Thus job resources can reduce job demands and stimulate personal growth, learning, and development (Bakker and Demerouti, 2007). In this case, job resources can buffer the impact of job demands on exhaustion, whereas job demands strengthen the motivating role of job resources. As a result, individuals could seek and increase resources to deal with the growing job demands, so as to balance job resource and job demand.

The important manifestation of modern business organizations is authorization and self-management. In the digital economy era, the economy is developing rapidly and on a large scale. The vigorous development of technological innovation, big data, cloud computing and artificial intelligence provide new impetus for value creation and employment in the new situation (Temelkova, 2018). Facing the rapidly changing environment and the increase of complex tasks, the organization needs to have greater flexibility to deal with a series of non-routine, non-repetitive, complex and challenging team tasks. A single person is unlikely to be able to Edelmann et al. (2020). Therefore, digital leaders need authorization, and encourage employees to participate in the decision-making process. This kind of leadership will effectively help organizations in expressing different ideas (Abbasi et al., 2020), and promoting employee job crafting. First, digital leadership can promote the integration of new digital technology into workplaces that promote employee job crafting. Specifically, to keep pace with the development of digital technology, employees will optimize work with new methods, so as to realize automation and intelligence of their work. Second, digital leadership provide employees with discretion and enhancing individuals’ motivation and sense of ability to perform tasks (Agarwal and Farndale, 2017). These management methods subverts the traditional form of highly centralized power and give individuals in the organization sufficient resources and autonomy to complete tasks in the digital environment. Additionally, digital leadership also helps enterprises make “real-time” decisions to quickly and flexibly deal with uncertainties and future challenges, which require individual to remodel their work to better fit the environment (Dahou and Hacini, 2018). Lastly, digital leadership can also improve employees’ job crafting by reducing employees’ work pressure. For example, they also help followers to make adjustments between work roles and personal value, and stimulate individuals’ trust in the organization (Ruiz-Palomo et al., 2020). High-quality digital leaders succeed in creating a shared sense of “us” in the organization, and this organizational identification in turn produces positive results and helps reduce work stress. All in all, digital leaders will stimulate employee job crafting through promoting the usage of new digital technology, providing the direction for the organization, making “real-time” decisions and helping employee adjust (Edelmann et al., 2020).

***Hypothesis 2:** Digital leadership is positively related to employee job crafting.*

### Mediating Effect of Employee Job Crafting Between Digital Leadership and Employee Creativity

Employee job crafting is a type of proactive or self-initiated and change-oriented behavior to ensure a better fit between the job and person (Tims et al., 2012), which will be influenced by leadership behavior and resources needed to complete the task. In the case of the digital age, employees are easily be exposed to pressure, physical and emotional exhaustion without sufficient resources needed for work. However, digital leadership, as a new leadership model in the digital era, can promote knowledge sharing, resource acquisition, mutual cooperation and continuous self-management among members in organizations. Consequently, digital leadership can better integrate culture and digital competence to utilize digital technology as part of leadership style to bring value to organizations and promote employee job crafting (Wasono and Furinto, 2018). On the one hand, digital leader will provide sufficient resources to help their subordinates effectively dealing with the job demands they face during job crafting, preventing high levels of exhaustion. On the other hand, digital leader can also optimize instead of reducing job demands to help employees reserving energy, thereby avoiding energy depletion (Petrou et al., 2015).

Employee job crafting also have positive effects to employees’ creativity. First, Job crafting behaviors may result in increased organizational commitment because through crafting, employees may improve the balance of challenges and demands to find a better fit (Tims et al., 2016). Second, individuals engage in job crafting to stimulate creativity, their fulfillment at work, positive work identity, work-related well-being, and job performance (Tims et al., 2012). Third, job crafting can trigger positive change attitudes through the meaning-making process that it stimulates. Finding meaning implies that individuals are able to understand what happens around them and link changes in their work environment to their own personal goals and values helping them to remain creative vitality (Demerouti et al., 2021). Thus, the following hypothesis is proposed.

***Hypothesis 3:** Employee job crafting mediates the relationship between digital leadership and employee creativity.*

### Moderating Role of Person-Organization Fit

Person-organization fit describes the interpersonal compatibility between individuals and members of their immediate work companies (Kristof-Brown and Stevens, 2001). Given the growing competition for talent among organizations, examining person-organization fit’s effect on individual behavior is urgent and necessary (Kristof-Brown et al., 2005; Swider et al., 2015). Values has been seen as the most suitable way of operationalizing fit, because values constitute a reliable guide to understanding a wide range of subsequent work attitudes and behavior (Chen et al., 2016). In line with previous research, we mainly focus on the congruence of employees’ values (Chen et al., 2016).

According to person-organization fit theory, an individual’s behavior results from the interaction between the person and the organization (Argyris, 1957). Person-organization fit can be categorized into two broad classes: supplementary fit and needs–supplies fit. Supplementary fit is typically assessed as similarity on psychological characteristics such as values, goals, attitudes, or personality traits. Complementary fit often refers to a person possessing the requisite knowledge, skills, and abilities to meet job demands (Muchinsky and Monahan, 1987; Kristof-Brown et al., 2014; Seong et al., 2015). Person-organization fit theory posits that organizations have characteristics that have the potential to be congruent with characteristics of individuals, and that individuals’ attitudes and behaviors will be influenced by the degree of congruence or “fit” between individuals and organizations (Argyris, 1957; Pervin, 1989). Consequently, a better person-organization fit has been linked to organizational attraction and retention, recruiters’ selection decisions, and employees’ work-related attitudes and proactive actions.

In the context of digitalization, person-organization fit has been argued to result in an increase in motivation, effort, energy, and persistence, as well as involvement with the organizational mission (Wang et al., 2011; Hamstra et al., 2019). When individuals’ values fits with the organization’s values, digital leaders hold the point that members in their company embrace similar values, thus enabling them to trust each other and communicate about important issues (Edwards and Cable, 2009). Such behavior transmit a signal that leaders think highly of their value (Nordin et al., 2019). Accordingly, employees will show more positive attitudes and behaviors, and have sufficient motivation to craft their job and achieve organizational goals. Under this situation, a higher person-organization fit helps digital leaders establish a more positive and harmonious relationship with their employees and gives employees the motivation of “should do” to craft the way they work, compared witha lower person-organization fit (Safavi and Bouzari, 2020). Analogously, a high fit with the organization will be beneficial to reduce employee turnover and improving the digital leaders willingness to help employee make adjustments between work roles and personal value, and stimulate employee job crafting (Hamstra et al., 2019). Consequently, the higher the person-organization fit, the higher leaders’ interactions with their employees in organization, and the greater the inspiration for knowledge sharing and resource inter-flow to promote employee job crafting (Hung et al., 2020).

***Hypothesis 4:** Person-organization fit moderates the relationship between digital leadership and employee job crafting, such that the relationship is stronger among organization with high person-organization fit than among those with low person-organization fit.*

### Moderated Mediating Effect

Hypotheses 4, respectively, illustrate a moderating effect of person-organization fit on the relationship of “digital leadership-job crafting.” According to the above discussions, this study integrates the job demands-resources model and person-organization fit theory to construct a moderating mediation model, which is based on the moderating mediator inference method (Edwards and Lambert, 2007). That is, person-organization fit positively moderates the mediating effects of digital leadership on employee creativity *via* employee job crafting. Thus, the following hypothesis is proposed.

***Hypothesis 5:** Person-organization fit positively moderates the indirect effect of digital leadership on employee creativity through employee job crafting. That is, the higher the level of person-organization fit, the greater the mediating effect of employee job crafting.*

**FIGURE 1:**
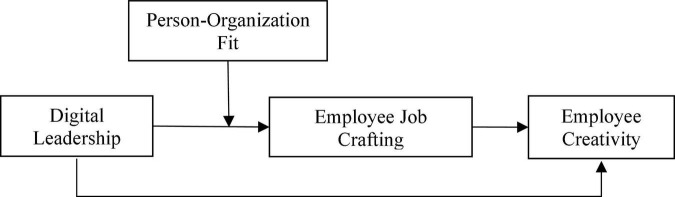
Theoretical model.

The theoretical model of this study is shown in Figure 1.

## Materials and Methods

### Sample and Procedures

This survey was conducted between April and May of 2021 with a web-based questionnaire. Participants were recruited in the following ways. Firstly, we sent the questionnaire to the companies’ employees by directly contacting the companies’ human resources supervisor, who further distributed the questionnaire to the employees. Second, we indirectly distributed questionnaires with the help of MBA students who worked in various Chinese companies.

The questionnaire survey was composed of two stages: In Time 1 (T1), employees complete questionnaires regarding a predictor variable (digital leadership), a moderating variable (person-organization fit), and demographic variables (age, gender, education, working seniority, and position). After a month, at Time 2 (T2), the same participants completed questionnaires regarding mediating variables (employee job crafting) and a dependent variable (employee creativity). To match the responses of T1 and T2, participants were asked to fill in the last four digits of their ID numbers in the questionnaire.

A total of 457 questionnaires were collected, and 100 were discarded for missing data, leaving 357 valid questionnaires and a response rate of 78.1%. Among the samples, 145 (40.6%) are males and 212 (59.4%) are females. In terms of age, 189 (52.9%) are below 30 years old, 135 (37.8%) are between 31 and 40 years old, 33 (9.2%) are over 41 years old. In terms of education, 67(18.8%) reach a junior college degree or below, 245 (68.6%) has a bachelor’s degree, and 45(12.6%) has a master’s degree or above. In terms of working seniority, 178(49.9%) answered less than 5 years, 121 (33.9%) for 6–10 years, and 58 (16.2%) for more than 11 years.

### Measures

All scales’ items were originally developed in English and were therefore translated into Chinese, and all scales’ items are measured on a five-point Likert scale from 1 = “strongly disagree” to 5 = “strongly agree.”

### Digital Leadership

Digital leadership was measured with the six-item scale developed by Zeike et al. (2019). The items are as follow: (1) my leader think using digital tools is fun; (2) my leader is a digital expert; (3) When it comes to digital knowledge, my leader is always up to date; (4) my leader driving the digital transformation forward proactively in our unit; (5) my leader can make others enthusiastic about the digital transformation; (6) my leader have a clear idea of the structures and processes that are needed for the digital transformation. Cronbach’s alpha for this scale was 0.852.

### Employee Job Crafting

Employee job crafting was measured with the 21-item scale developed by Tims et al. (2012). We use three dimensions (increasing job resources, increasing challenging job demands and decreasing hindering job demands) with and 15 items. Considering repetition in the translation, three items are deleted and the scale with 12 items was used. The items were as follows: (1) I try to develop my capabilities; (2) I try to develop myself professionally; (3) I try to learn new things at work; (4) I make sure that I use my capacities to the fullest; (5) I decide on my own how I do things; (6) I make sure that my work is mentally less intense; (7) I try to ensure that my work is emotionally less intense; (8) I manage my work so that I try to minimize contact with people whose problems affect me emotionally; (9) I organize my work to minimize contact with people whose expectations are unrealistic; (10) when an interesting project comes along, I offer myself proactively as project co-worker; (11) when there is not much to do at work, I see it as a chance to start new projects; (12) I regularly take on extra tasks even though I do not receive extra salary for them. Cronbach’s alpha for this scale was 0.871.

### Person-Organization Fit

Person-organization fit was measured with the five-item scale developed by Resick et al. (2007). The items were as follows: (1) I feel my values “match” or fit this organization and the current employees in this organization; (2) I think the values and personality of this organization reflect my own values and personality; (3) the values of this organization are similar to my own values; (4) my values match those of current employees in this organization; (5) I feel my personality matches the “personality” or image of this organization. Cronbach’s alpha for this scale was 0.827.

### Employee Creativity

Employee creativity was measured with the four-item scale developed by Baer and Oldham (2006). The items were as follows: (1) I suggests many creative ideas that might improve working conditions at the organization; (2) I often comes up with creative solutions to problems at work; (3) I suggests new ways of performing work tasks; (4) I am a good source of creative ideas. Cronbach’s alpha for this scale was 0.821.

### Control Variables

Previous literature has shown that demographic variables and team characteristic variables may influence employee creativity, including age, gender, education, and working seniority. Thus, these variables are controlled in this study. Gender is measured as a dummy variable (1 = male, 2 = female). Age is divided into three levels (1 = under 30 years, 2 = 31–40 years, 3 = over 41 years). Education is divided into three levels (1 = junior college or below, 2 = bachelor’s degree, 3 = master’s degree or above). Working seniority is divided into three levels (1 = less than 5 years, 2 = 6–10 years, 3 = more than 11 years).

## Results

### Reliability and Validity

The Cronbach’s alpha of digital leadership, job crafting, person-organization fit, individual creativity were 0.852, 0.871, 0.827, and 0.821, all of which were greater than the critical value of 0.7. The results of Cronbach’s alpha indicated that the questionnaire has good reliability. MPLUS8.0 was used to carry out the CFA. Compared with other competition models, the theoretical four-factor model (digital leadership, employee job crafting, person-organization fit, individual creativity) had a better fit to the data [χ2/df = 2.14, comparative fit index (CFI) = 0.902, Tucker–Lewis index (TLI) = 0.911, root mean squared error of approximation (RMSEA) = 0.057, standardized root mean square residual (SRMR) = 0.048] (see Table 1). The results of CFA showed that the theoretical four-factor model had satisfactory discriminant validity.

**TABLE 1:** Results of confirmatory factor analysis.

	Factor	χ^2^	df	χ^2/^df	RMSEA	TLI	CFI	SRMR
Four-factor model	DL, JC, POF, IC	681.590	318	2.14	0.057	0.911	0.902	0.048
Three-factor model	DL, JC+POF, IC	769.122	321	2.40	0.063	0.891	0.880	0.052
Two-factor model	DL+JC+POF, IC	957.316	323	2.96	0.074	0.845	0.832	0.057
One-factor model	DL+JC+POF+IC	1051.125	324	3.24	0.079	0.822	0.808	0.060
Unmeasured latent method factor model		682.756	320	2.13	0.056	0.911	0.903	0.049

*N = 357; DL, digital leadership; JC, employee job crafting; POF, person-organization fit; EC, employee creativity.*

### Common Method Variance

Although the anonymous measurement method and two-wave design in a survey were used to reduce common method variance (CMV) in the data collection. However, CMV may still occur because all variables were measured by individual self-evaluation. Thus, the Harman single-factor test was used to assess the existence of CMV. The results showed that the first factor solution in the exploratory factor analysis indicated only explained 38.29% (<50%) loading, which proved the absence of CMV (Woszczynski and Whitman, 2004). Further, we conducted the unmeasured latent method factor, that all items were loaded on both this latent method factor and trait factors (Podsakoff et al., 2003), to test CMV. A comparison of the latent method factor model (χ2/df = 1.797, CFI = 0.938, TLI = 0.934, RMSEA = 0.044, SRMR = 0.041) and the theoretical five-factor model (χ2/df = 1.794, CFI = 0.938, TLI = 0.934, RMSEA = 0.044, SRMR = 0.041) indicated no significantly changes in CFI (Cheung and Rensvold, 2002). Thus, CMV should not be a severe problem in our study.

### Descriptive Statistics and Correlation Analysis

Table 2 shows the results of descriptive statistics and correlation analysis. The results indicate that digital leadership is positively correlated to job crafting (*r* = 0.610, *p* < 0.01), employee creativity (*r* = 0.664, *p* < 0.01). Job craft is positively correlated to individual creativity (*r* = 0.707, *p* < 0.01). The correlation between the mainly key variables provides the initial support to direct effect and indirect effect for our hypotheses.

**TABLE 2:** Means, standard deviations (SD), and correlations.

Variables	Mean	SD	Gender	Age	Education	Seniority	DL	JC	POF	EC
Gender	1.59	0.492	1							
Age	1.56	0.657	–0.029	1						
Education	1.94	0.557	–0.071	−0.250**	1					
Seniority	1.66	0.741	–0.098	0.747**	−0.159**	1				
DL	3.85	0.688	–0.076	0.064	–0.056	0.201**	1			
JC	4.08	0.469	–0.043	0.037	–0.044	0.146**	0.610**	1		
POF	4.03	0.602	−0.128*	0.088	0.024	0.238**	0.568**	0.612**	1	
EC	3.92	0.705	–0.091	0.107*	−0.123*	0.196**	0.664**	0.707**	0.638**	1

N = 357; **p < 0.01; *p < 0.05; DL, digital leadership; JC, employee job crafting; POF, person-organiztion fit; EC, employee creativity.

### Hypotheses Test

This study uses hierarchical regression method to test the direct effect of digital leadership on employee job crafting and employee creativity, as well as the direct effect of employee job crafting on employee creativity; Bootstrapping method was used to test the mediating role of employee job crafting between digital leadership and employee creativity, and the moderated mediating effect.

Hierarchical regression results are shown in Table 3. Hypothesis 1 proposed that digital leadership is positively associated with employee job crafting. As show in Table 3, after controlling employees’ gender, age, education, position level, and seniority, digital leadership is positively and significantly related to employee job crafting (β = 0.617, *p* < 0.001, model 5). Hypothesis 1 is therefore supported. Digital leadership is positively and significantly related to employee job crafting (β = 0.584, *p* < 0.001, model 2), therefore, hypothesis 2 is supported. According to model 6, when digital leadership and employee job crafting are included in the regression equation at the same time to predict employee creativity, the regression coefficient of digital leadership and employee creativity is still significant and positive (β = 0.345, *p* < 0.001, model 6) and less than that in model 5. At the same time, employee job crafting is positively and significantly related to employee creativity (β = 0.466, *p* < 0.001, model 6). Hypothesis 3 is supported. In addition, this study also adopts the bootstrapping method to test the mediating effect. Based on 5,000 repeated sampling tests, the results show that job crafting plays a significant mediating role between digital leadership and employee creativity. The indirect effect of “DL→JC→EC” is significant (β = 0.279, *p* < 0.001), and the 95% CI is [0.201, 0.374]. Thus, hypothesis 3 is further supported.

**TABLE 3:** Results of hierarchical regression.

	Employee job crafting			Employee creativity		
		
	Model 1	Model 2	Model 3	Model 4	Model 5	Model 6
Gender	–0.003	0.014	0.055	–0.046	–0.027	–0.034
Age	−0.188*	–0.059	–0.013	–0.135	0.001	0.029
Education	–0.062	–0.020	–0.028	−0.138**	−0.094*	−0.084*
Exp	0.198*	0.038	–0.028	0.159*	–0.010	–0.028
Position	−0.209**	–0.089	–0.021	−0.296**	−0.169***	−0.128**
DL		0.584***	0.258***		0.617***	0.345***
JC						0.466***
POF			0.397***			
DL*POF			0.199***			
F	5.482***	35.789***	46.397***	10.757***	53.051***	78.213***
R^2^	0.072	0.380	0.516	0.133	0.476	0.611
ΔR^2^		0.308	0.136		0.343	0.135

N = 357; ***p < 0.001; **p < 0.01;*p < 0.05; DL, digital leadership; JC, employee job crafting; POF, person- organization fit; EC, employee creativity.

**FIGURE 2:**
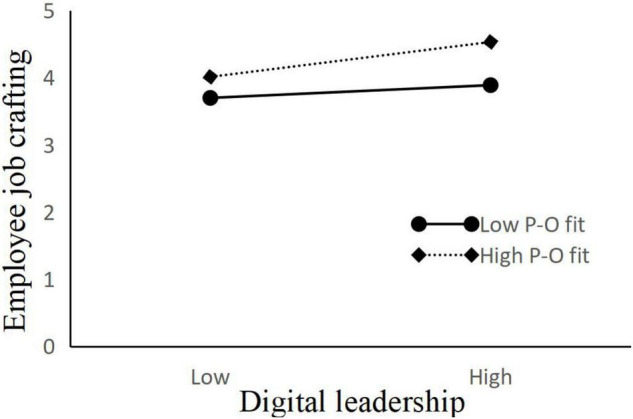
Moderating effect of person-organization fit on the relationship of digital leadership and employee job crafting.

We examine the moderating effect of person–organization fit between the relationship of digital leadership and employee job crafting. As shown in Table 3, the interaction of digital leadership and person–organization fit is significantly and positively related to employee job crafting (β = 0.199, *p* < 0.001, model 3), indicating that person–organization fit positively moderates the relationship of digital leadership and employee job crafting. According to the suggestions of Aiken et al. (1991), this study further inspected the moderating effect by testing the simple slopes at high and low levels of person–organization fit, and the moderating effect diagram is drawn according to the regression coefficient (Figure 2).

For the moderated mediating effect test, a “mediating effect difference test” is used. By adding or subtracting one SD from the mean value of person-organization fit, the conditional mediating effects of employee job crafting under high and low person-organization fit are formed and compared for significance at different levels. If the CI excludes zero, the mediated mediating effect is significant. Table 4 shows the results. The model of digital leadership influencing employee creativity *via* employee job crafting shows that at low levels of person-organization fit, the mediating effect of job crafting is not significant (β = 0.101, *p* < 0.055, the CI is [0.012, 0.221], excluding zero). At high levels of person-organization fit, the mediating effect of employee job crafting is significant (β = 0.266, *p* < 0.001, the CI is [0.175, 0.387], excluding zero). The two groups show significant differences (β = 0.101, *p* < 0.05, the CI was [0.029, 0.282], excluding zero). These results show that the indirect effect between digital leadership and employee creativity is significantly greater than that of low person-organization fit. Thus, Hypothesis 5 is confirmed.

**TABLE 4:** Results of moderated mediating effect test (DL→JC→EC).

Person-organization fit	Estimate	S.E.	Est./S.E.	*P*-value	95% CI	
					
					Lower limit	Upper limit
High (+1SD)	0.266	0.054	4.953	0.000	0.175	0.387
Low (-1SD)	0.101	0.053	1.916	0.055	0.012	0.221
Differences	0.165	0.064	2.589	0.010	0.029	0.282

*DL, digital leadership; JC, employee job crafting; POF, person-organization fit; EC, employee creativity. The difference is equal to the mediating effect of conditions under high person-organization fit minus the mediating effect of conditions under low person-organization fit.*

## Discussion

The past few years have witnessed growing academic interest in digital leadership (e.g., El Sawy et al., 2016; Larjovuori et al., 2016; Gierlich-Joas et al., 2020; Klein, 2020; Bresciani et al., 2021; Claassen et al., 2021; de Araujo et al., 2021), but few studies have focused on the relationship between digital leadership and employee creativity. Based on the job demands–resources model and person–organization fit theory, this study examines the relationship between digital leadership and employee creativity. The results of the empirical study support the proposed research model, and the main findings are as follows:

First, digital leadership is positively related to employee creativity. The more digital leadership shown by the leader, the more effective it is in stimulating employee creativity. This findings agree with previous researches holding that digital leadership plays an important role in promoting employee creativity (Wasono and Furinto, 2018; Mihardjo et al., 2019).

Second, employee job crafting mediates the relationship between digital leadership and employee creativity. When enterprise leaders show digital leadership, job crafting of employees improves, stimulating their creativity. On the one hand, digital technology will improve the flexibility of the organization and the utilization of resources to some extent by empowerment (Abbasi et al., 2020). On the other hand, digital technology has changed the traditional way of work (Gilson et al., 2015). Therefore, employees will make job crafting to enable their abilities to fit their jobs, and then lead to enhance their creativity.

Third, person-organization fit positively moderates the relationship between digital leadership and job crafting. Person–organization fit theory posits that organizations have characteristics that have the potential to be congruent with characteristics of individuals and that individuals’ attitudes and behaviors will be influenced by the degree of congruence or “fit” between individuals and organizations (Argyris, 1957; Pervin, 1989). Compared with low person-organization fit, the positive relationship between digital leadership and employee job crafting is stronger under high person-organization fit. Furthermore, person-organization fit positively moderate the indirect effect of digital leadership on employee creativity through employee job crafting.

### Theoretical Contributions

First, this study examined the relationship between digital leadership and employee creativity. Leadership has always been regarded as a key antecedent variable to predict organizational and individual behavior. Previous studies have explored the impact of different leaders on creativity. For example, in a recent meta-analysis, 13 leadership types (transformational, transactional, ethical, humble, leader-member exchange, benevolent, authoritarian, entrepreneurial, authentic, servant, empowering, supportive, and destructive) were examined using data from 266 studies (Lee et al., 2020). The results show that almost all those 13 leadership types are modestly correlated with employee creativity or employee innovation behavior. This findings provides some enlightenment for our research. In the digital economy era, digital technology has significantly changed our workplace and the way we do business. Accordingly, a large number of office applications are beginning to emerge and were adopted by organizations. With the help of these applications, leaders can establish remote workplaces and virtual teams to complete tasks by independence from a given time and place as well as changing work demands in general (Gilson et al., 2015). Moreover, the emergence of WeCom, DingTalk, and E-mail increasingly simplifies the team’s communication and knowledge sharing among employees, and promotes the autonomy, flexibility, and creativity of the team. But for leaders, how to manage decentralized employees and promote employees’ creativity is very important. Few studies have focused on digital leadership as a key antecedent to predict employee creativity. This study is a step toward filling this gap by exploring the relationship between digital leadership and employee creativity. The results show that digital leadership does improve employees’ creativity, enriching the literature on antecedents of employee creativity.

Second, this study examined the mediating role of employee job crafting between digital leadership and employee creativity. Previous literature shows that leadership mostly indirectly affects employees’ creativity, rather than directly affecting employees’ creativity. Unfortunately, few studies have focused on the mediating mechanism of digital leadership on employees’ job crafting. Based on the job demands-resources model, we propose that employee job crafting will mediate the relationship between digital leadership and employee creativity. First, digital transformation will bring more opportunities for employees to obtain the information and resources which are needed to complete their tasks. Secondly, digital transformation puts forward new requirements for employees’ working methods. Employees need to balance the resources and demands in their work to better complete their work tasks and reduce their psychological insecurity. Therefore, in this study, we take employee job crafting as a mediator between digital leadership and employee creativity. The findings confirmed that digital leadership positively relate to employee creativity *via* employee job crafting. The findings supplements the literature on digital leadership by exploring how digital leadership affects employee creativity.

Third, this study extends the boundary conditions under which the mediating effect of employee job crafting between digital leadership and employee creativity are strengthen or weaken. To our knowledge, no existing study has explored the moderating mechanism of digital leadership on employees’ job crafting. According to person-organization fit theory, organizations have characteristics that have the potential to be congruent with characteristics of individuals, and individuals’ attitudes and behaviors will be influenced by the degree of congruence or “fit” between individuals and organizations (Argyris, 1957; Pervin, 1989). Consequently, a better person-organization fit has been positively linked to organizational attraction and retention, recruiters’ selection decisions, and employees’ work-related attitudes and proactive actions. The findings show that person-organization fit can moderate the relationship between digital leadership and employees’ job crafting. When there is higher matching between individuals and organizations, individuals can better understand leaders’ behavior and show their support to their leaders. Therefore, individuals will turn this recognition into the impetus of work, and then improve personal enthusiasm and initiative to improve the behavior of job crafting. In this study, we also found that person-organization fit also moderates the mediating role of job crafting between digital leadership and employee creativity. The findings suggest that higher person-organization fit could strengthen the relationship between digital leadership and employee job crafting. It provides an answer to the question of when digital leadership affects employee job crafting, and deepens the understanding of the boundary conditions of digital leadership affecting employee creativity through employee job crafting.

### Practical Implications

Digitalization has become an irresistible and irreversible trend of enterprises. The digital age is an era characterized by VUCA (volatility, uncertainty, complexity, and ambiguity). In China, with the booming development of a new wave of scientific and technological revolution and industrial reform, the new advantages of the digital economy are important in realizing quality, efficiency, and power reform, becoming essential to promoting high-quality integrated development. Therefore, the research on digital leadership has practical implications.

First, given the key role of digital leadership in promoting employee creativity, enterprises should pay more attention to and cultivate digital leadership. Digital leadership can provide vision and direction for enterprises, and lead them to a promising future. However, how to cultivate digital leaders can start from the following three aspects. First, effective procedures and standards can be established to select and promote leaders with digital capabilities. Second, organizations can carry out corresponding leadership training courses and development projects to advocate for leaders’ digital ability. Third, relevant assessment, reward, and punishment systems can be formulated to provide more support for digital leaders, to encourage the improvement of leaders’ digital ability.

Second, organizations should pay attention to the fit of organizational climate and employees’ values. The findings show that person–organization fit positively moderates the mediating effect of employee job crafting between digital leadership and employee creativity. Consequently, supplementary and complementary fit are the key issues on which organizations should focus. On the one hand, organizations need to attach importance to the needs of employees and help employees to make plans for their future development. This behavior enables employees to be more clear about their role positioning and then increase their organizational identity. On the other hand, organizations can create an inclusive atmosphere and encourage a diverse organizational culture. Given that individuals have different characteristics, an inclusive climate facilitates employees to create a collective perception of “we” within the organizations. As a result, the value fit between individuals and organizations will be improved.

Third, organizations should be aware of employees’ job crafting. Managing employee job crafting behaviors that contribute to personal and organizational goals is the of a manager. Therefore, managers could inform their subordinates about job crafting strategies and stimulate them to take job crafting behaviors when they desire more challenging work or less hindering job demands (Petrou et al., 2016). In addition, managers need to pay more attention to the needs of their subordinates in relation to their resources, challenges, and hindrances (Wingerden et al., 2015).

### Limitations and Future Research

First, we collect data at different time points that will avoid the problem of CMV, and reflect the causal relationship of variables in time to some extent. However, all variables in this study are employee self-report, which may lead to common method variance. Therefore, future research can use multi-time points and multi-source methods to collect data. In addition, more rigorous experimental design (such as matched-pair study, longitudinal design study and experimental method) can be adopted.

Second, this study examines the relationship between digital leadership and employee creativity at the individual level. Future research can test the relationship between digital leadership and creativity from a multi-level perspective. In addition, this study is conducted in China which is regarded as high power distance culture. Thus, researchers can study the relationship in different countries to obtain more convincing and generalized results.

Third, by integrating the demands-resources model and person-organization fit theory, this study examine the mediating role of employee job crafting between digital leadership and employee creativity, and the moderating role of person-organization fit between digital leadership and employees’ job crafting. Future research can explore the boundary between digital leadership and creativity based on different perspectives and theories.

## Data Availability Statement

The raw data supporting the conclusions of this article will be made available by the authors, without undue reservation.

## Ethics Statement

As protection of all participants, all subjects read informed consent before participating in this study and voluntarily made their decision to complete surveys. The protocol was approved by an institutional review board in Xiangtan University of China.

## Author Contributions

JZ developed the theoretical model, collected the data, and wrote the manuscript. BZ collected the data, analyzed the data, and participated in the manuscript writing. QC participated in the manuscript writing. MX participated in revising the manuscript. All authors contributed to the article and approved the submitted version.

## Conflict of Interest

The authors declare that the research was conducted in the absence of any commercial or financial relationships that could be construed as a potential conflict of interest.

## Publisher’s Note

All claims expressed in this article are solely those of the authors and do not necessarily represent those of their affiliated organizations, or those of the publisher, the editors and the reviewers. Any product that may be evaluated in this article, or claim that may be made by its manufacturer, is not guaranteed or endorsed by the publisher.
